# TOXPANEL: A Gene-Set Analysis Tool to Assess Liver and Kidney Injuries

**DOI:** 10.3389/fphar.2021.601511

**Published:** 2021-02-09

**Authors:** Patric Schyman, Zhen Xu, Valmik Desai, Anders Wallqvist

**Affiliations:** ^1^ DoD Biotechnology High Performance Computing Software Applications Institute, Telemedicine and Advanced Technology Research Center, U.S. Army Medical Research and Development Command, Fort Detrick, MD, United States; ^2^ The Henry M. Jackson Foundation for the Advancement of Military Medicine, Inc., Bethesda, MD, United States

**Keywords:** predictive toxicology, systems toxicology, toxicogenomics, nephrotoxicity, hepatotoxicity, RNA-seq

## Abstract

Gene-set analysis is commonly used to identify trends in gene expression when cells, tissues, organs, or organisms are subjected to conditions that differ from those within the normal physiological range. However, tools for gene-set analysis to assess liver and kidney injury responses are less common. Furthermore, most websites for gene-set analysis lack the option for users to customize their gene-set database. Here, we present the ToxPanel website, which allows users to perform gene-set analysis to assess liver and kidney injuries using activation scores based on gene-expression fold-change values. The results are graphically presented to assess constituent injury phenotypes (histopathology), with interactive result tables that identify the main contributing genes to a given signal. In addition, ToxPanel offers the flexibility to analyze any set of custom genes based on gene fold-change values. ToxPanel is publically available online at https://toxpanel.bhsai.org. ToxPanel allows users to access our previously developed liver and kidney injury gene sets, which we have shown in previous work to yield robust results that correlate with the degree of injury. Users can also test and validate their customized gene sets using the ToxPanel website.

## Introduction

ToxPanel is a web-based tool to assess liver and kidney injury from *in vitro* or *in vivo* genomic data. In the field of toxicogenomics, a common assumption is that toxicity is associated with a change in the expression of either a single gene or a set of genes (i.e., a module or a gene signature) (Hamadeh et al., 2002; Segal et al., 2004; Fielden et al., 2005; Minowa et al., 2012; Sahini et al., 2014; Ippolito et al., 2015; Parmentier et al., 2017; Sutherland et al., 2019; Wang et al., 2019). Using a toxicogenomic approach, we previously derived 11 liver- and 8 kidney-injury modules (Te et al., 2016) from the Open Toxicogenomics Project-Genomics Assisted Toxicity Evaluation System (TG-GATEs) database (Igarashi et al., 2015), where each injury module is uniquely associated with a specific organ-injury phenotype, see Table 1. The TG-GATEs database contains gene-expression data from Sprague Dawley rats exposed to different chemicals for 4–29 days with corresponding documented and graded histopathological injury phenotypes.

**TABLE 1:** List of liver and kidney injury modules grouped into general classes with the number of genes in each module.

	Inflammation		Degeneration		Proliferation	
Liver	Fibrogenesis	48	Anisonucleosis	65	Bile duct proliferation	16
	Cellular infiltration	25	Nuclear alteration	111	Oval cell proliferation	126
	Hematopoiesis	27	Cytoplasmic alteration	18	Cellular foci	35
	Single cell necrosis	11	Granular degeneration	18		
Kidney	Necrosis	18	Degeneration	65		
	Fibrogenesis	125	Dilatation	8		
	Cellular infiltration	42	Inclusion bodies (cytoplasmic)	40		
			Casts (hyaline)	23		
			Hypertrophy^a^	16		

^a^ Hypertrophy can also be the result of proliferation.

With the use of TG-GATE, we identified common gene responses (injury modules) that correlated with the severity of injury, including fibrosis, using in silico approaches. In Table 1 we summarized the injury modules we identified in previous studies (Te et al., 2016). For a biological interpretation, we categorized the histological endpoint into their pathological responses, inflammation, degeneration, and proliferation. The gene module approach outperforms individual genes in predicting severity of histological damage (AbdulHameed et al., 2014; Tawa et al., 2014; Te et al., 2016; Schyman et al., 2020b).

Adverse outcome pathway (AOP) is a recent development in toxicology that emphasize a mechanism-based approach to toxicological evaluation as an aid in developing alternatives to animal testing (Ankley et al., 2010). It typically summarizes complex toxicological phenotype in a flow chart-like diagram consisting of molecular initiating events (MIE), key events (KE), and adverse outcomes (AO) (Vinken, 2013). This type of mechanistic outline allows for the development of new *in vitro* tests that captures the adverse outcome caused by *in vivo* chemical exposures (Kleinstreuer et al., 2018). We and others have shown that gene expression data can be used to gain insights into the key events of an AOP at a molecular-level (Oki et al., 2016; AbdulHameed et al., 2019). The modules listed in Table 1 represent gene sets that have been associated with adverse outcome. The focus of current paper is on the development of a web-based tool that will allow any user to access and evaluate the activation of these gene modules for their own data. The output from ToxPanel can also be construed as a molecular-level read out for activation of key event in adverse outcome pathway. Our injury modules complement Wiki-AOPs as they offer an interpretation of an adverse biological response that is non-chemical specific. However, they do not offer detail mechanistic insights, which KEGG pathways or wiki-pathways can provide (Kanehisa and Goto, 2000; Martens et al., 2020). We have shown that the combination of our modular approach to identify key injury phenotype together with pathway analysis, provided in ToxPanel, can be useful when understanding the underlying molecular mechanisms in e.g., liver or kidney injury (Schyman et al., 2020a; Schyman et al., 2020b).

**FIGURE 1:**
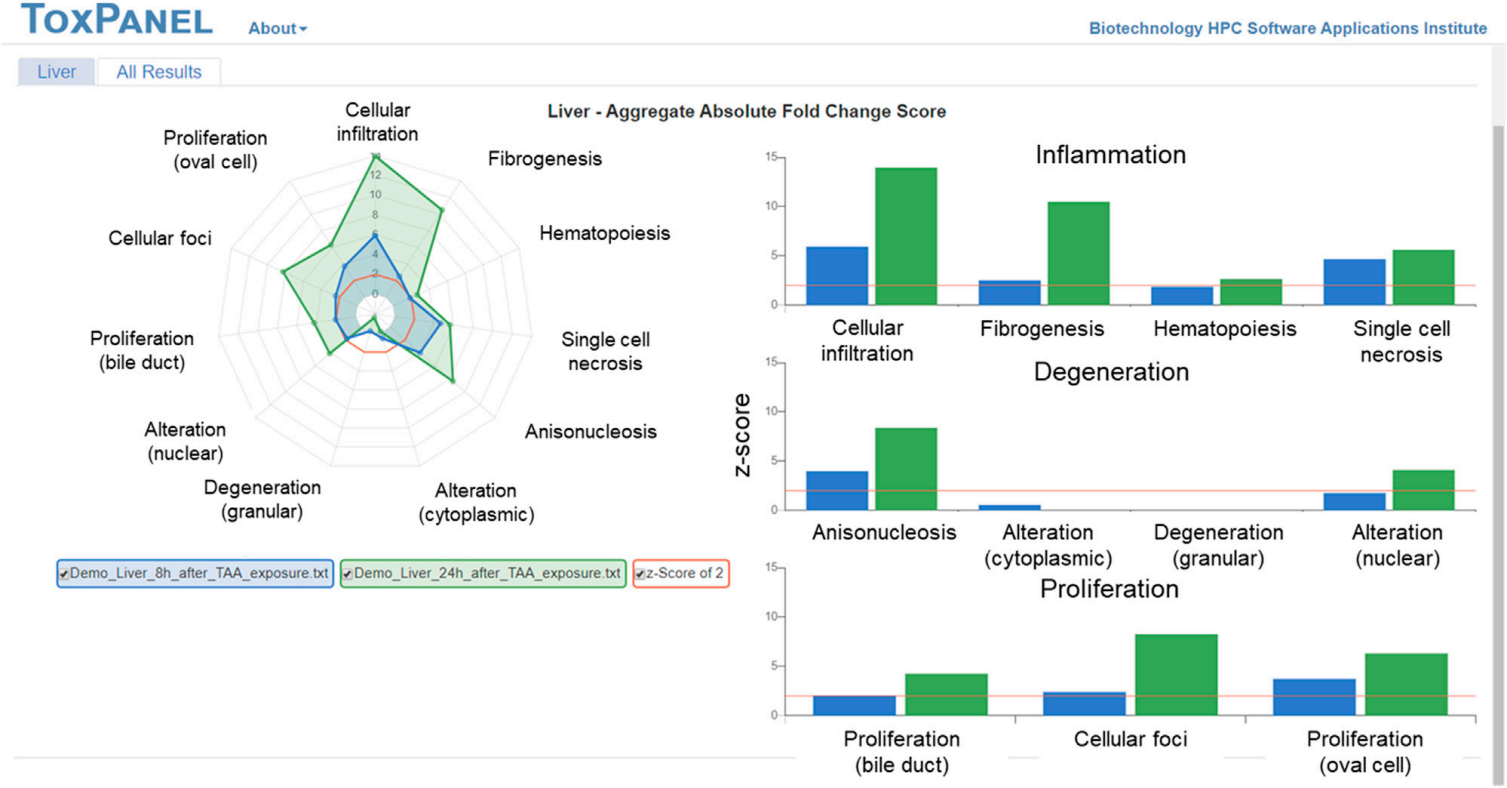
Gene-expression changes in the rat liver 8 and 24 h after thioacetamide (TAA) [100 mg/g] exposure. The right panel shows a strong inflammatory response 24 h after TAA exposure with *cellular infiltration* and *fibrogenesis* as primarily liver-injury phenotypes.

We previously validated these injury modules *in vivo* by treating Sprague Dawley rats with thioacetamide (Schyman et al., 2018), an organosulfur compound extensively used in animal studies as a fibrosis-promoting liver toxicant. Our ToxPanel approach correctly identified *cellular infiltration* and *fibrogenesis* as primarily liver-injury phenotypes induced by thioacetamide (Figure 1). Figure 1 shows the increased injury module activations over time related to inflammation and proliferation in accord with the progression of the fibrosis injury phenotype.

Furthermore, we have found that our injury modules can predict *in vivo* injury endpoints from *in vitro* RNA sequence (RNA-seq) data with a strong correlation (*R* ^2^ > 0.6) (Schyman et al., 2019). In this study we compared *in vivo* rat data with *in vitro* cellular data 24 h after treatment of thioacetamide. The-top ranked liver-injury modules identified by our *in vitro* studies agreed with those identified *in vivo* using thioacetamide, indicating that *in vitro* cell injury was also associated with changes in the expression levels of fibrogenic genes.

Analysis of gene sets typically involves the use of tools for the enrichment analysis of specific biological pathways in gene annotation databases, such as KEGG (Kanehisa and Goto, 2000) and GO terms (The Gene Ontology Consortium, 2018). Pathway enrichment analysis tools are readily accessible in many widely used web applications, such as GSEA (Subramanian et al., 2005) and DAVID (Huang et al., 2008). An alternative approach involves analyzing *activation scores* derived from the aggregated fold-change (FC) values of the genes in a gene set or pathway and comparing it to a background set of FC values. Although this gene-set activation approach provides robust results (Ackermann and Strimmer, 2009; Yu et al., 2017), it is not available in most web applications.

**FIGURE 2:**
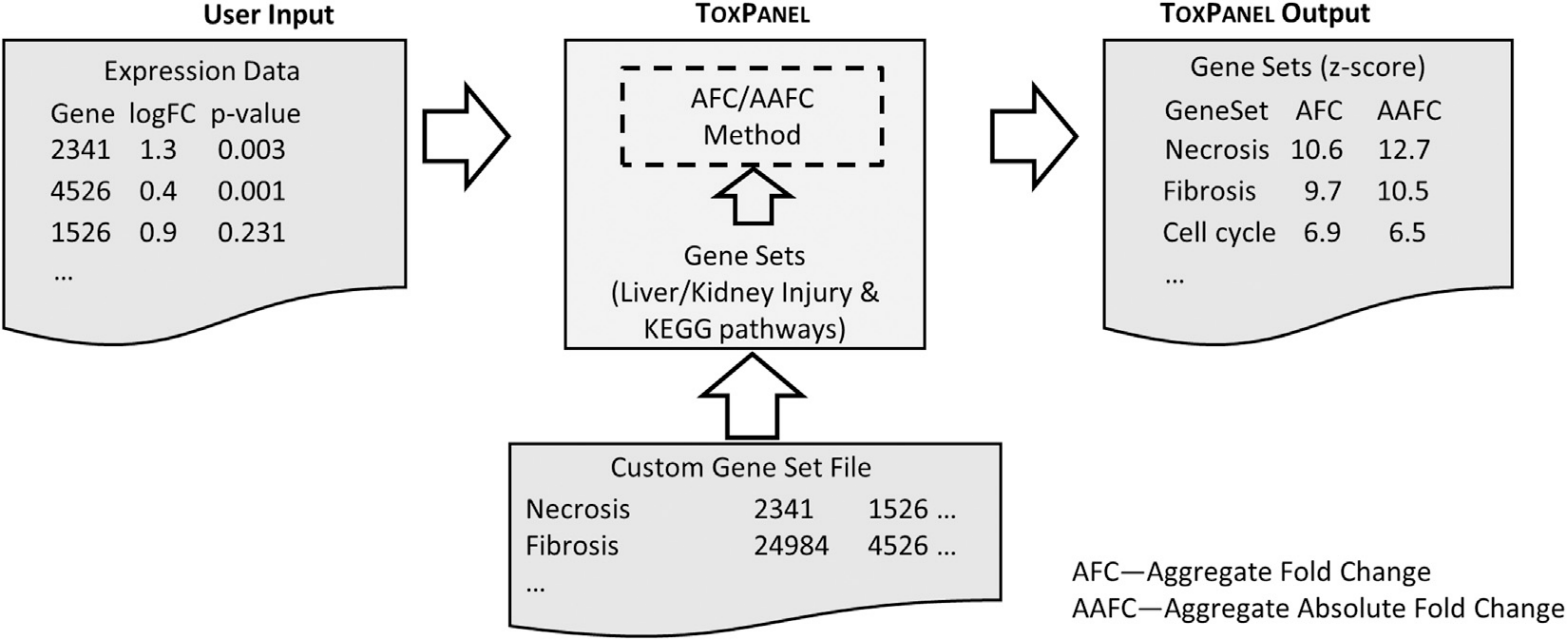
Schematic illustration of typical User Input and the optional Custom Gene Set file formats. The ToxPanel Output presents the calculated AFC and AAFC values for each gene set based on the log FC values in the User Input file.

Here, we present a web application that uses two gene-set activation methods, which we denote as aggregated FC (AFC) and aggregated absolute FC (AAFC). These methods are not limited to FC values *per se*, as they can also accept beta-values from Kallisto-Sleuth output (Bray et al., 2016; Pimentel et al., 2017) or z-score values as inputs. Figure 2 outlines a schematic image of ToxPanel‘s input and output files. AAFC and AFC can be used for predefined or custom-designed gene sets. In the application, the current default gene sets for these methods are liver- and kidney-injury modules, which are gene sets associated with specific injury phenotypes, such as liver fibrosis and kidney necrosis (Ippolito et al., 2015; AbdulHameed et al., 2016; Te et al., 2016; Schyman et al., 2018; Schyman et al., 2019; Wang et al., 2019; Schyman et al., 2020a; Schyman et al., 2020b). We also offer access to the rat and human KEGG pathways, as determined using Entrez gene IDs (Maglott et al., 2011). The gene-set format is compatible with MSigDB (Liberzon et al., 2011) and can be uploaded to the ToxPanel website for analysis. In a recent study in rats, we showed that our injury modules could link genomic responses to observed organ injuries (Schyman et al., 2018; Schyman et al., 2020a), demonstrating the promise of the modular approach in predicting rat *in vivo* results from rat and human *in vitro* genomic responses (Schyman et al., 2019; Schyman et al., 2020b).

## Methods

### Aggregated Fold-Change Activation

Detailed descriptions and performance characteristics of the aggregated fold change (AFC) activation method can be found in the original literature (Ackermann and Strimmer, 2009; Yu et al., 2017). In this method, we define the gene-set or KEGG pathway score as the sum of the *log*-transformed FC values of all genes in the set or pathway. We then use the pathway scores to perform null hypothesis tests and estimate the significance of each pathway by its *p*-value, defined as the probability that the pathway score for a random data set is greater than the score from the actual data set. The z-score is the number of standard deviations by which the actual gene-set value differs from the mean of randomly selected FC values (10,000 times). The sign of the gene-set score represents the direction of regulation: we consider the pathway up-regulated (overexpressed genes) if the net sum of the gene-expression levels after treatment is increased relative to control and down-regulated (suppressed genes) if it is decreased.

### Aggregated Absolute Fold-Change Activation

We recently used the aggregated absolute fold-change (AAFC) activation method to calculate the activation score of a gene set (Schyman et al., 2018; Schyman et al., 2019). This method identifies gene sets that are significantly changed or disrupted without considering the direction of change. The method, which takes the absolute values of the *log*-transformed FC values, performs well in identifying significantly altered pathways (Ackermann and Strimmer, 2009). Its potential shortcoming is that it disregards information about the direction of change in a pathway (whether it is up- or down-regulated i.e., if the sum of the activation scores of genes in a pathway increases or decreases relative to control).

The AAFC method first reads a list of gene FC values uploaded by the user and takes the absolute value of the *log*-transformed FC value for each gene. For each gene set, it then sums all of the absolute values to calculate the total absolute FC value. Subsequently, we use the gene-set scores to perform null hypothesis tests and estimate the significance of each gene set by its *p*-value, defined as the probability that the score for randomly selected FC values (10,000 times) is greater than the score from the actual gene set. A small *p*-value implies that the gene-set value is significant. As in the AFC method, the z-score is the number of standard deviations by which the actual gene-set value differs from the mean of the randomly selected FC values (10,000 times). The AAFC method, however, considers only positive z-score values, as negative z-score values indicate FC values smaller than the average absolute FC value.

### Implementation of the Web-Application

The ToxPanel web-application is delivered through encrypted Hypertext Transfer Protocol Secure (HTTPs) and can be accessed at toxpanel.bhsai.org. The implementation of ToxPanel consists of controller, database, and front view. The controller is written in Java and runs in JDK 1.8. The controller handles interaction with the user from file uploading to job submission. When submitting a job, the controller stores a record in the database and queue the job, which will run an R script for the analysis. After completing the job, the controller stores the result and notify the user through email. On the database side, PostgreSQL 10.5 is employed to provide sufficient data storage and retrieval capability. The front view is implemented with PrimeFace 7.0 library and BootsFaces 1.3.0 library with decoration of ChartJS 2.9.3 and customized Cascading Style Sheets (CSS). The two libraries provide convenient syntax and a wide range of user interface components. They serve as the backbone for the web user interface. The ChartJS 2.9.3 provides more advanced chart drawing and allows further tuning. The web service runs on Tomcat 8.5, which resides inside a docker container. This allows a speedy recovery if the web service ever encounters critical failure.

Upon visiting the site, the user is directed to the login page. The user can either login with a registered account or login as guest. The guest account is primarily for demonstration purpose, but all features are available. Once logged in, the user can upload gene expression data, specify job variables, and submit a job. The job will be queued and once completed the user can visit the result page through the history table.

## Results and Discussion

**FIGURE 3:**
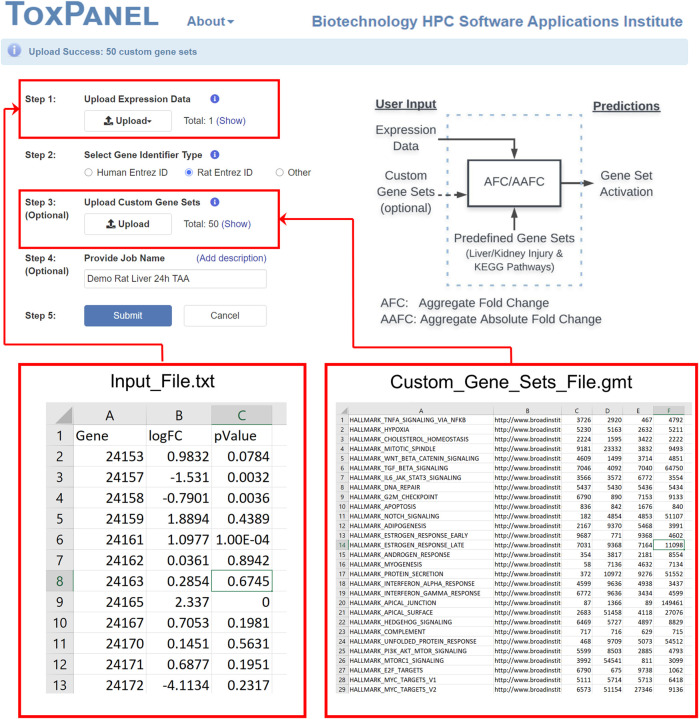
The figure illustrates the two input file formats for Step 1 and Step 3. The Step 1 input file with the gene expression data is required, but the *p*-values in column C are optional. The Step 3 Custom Gene Sets file is optional if one uses Human or Rat Entrez ID but required if Other gene IDs are used. The Custom Gene Sets format follows the .gmt format illustrated in the figure.

The main purpose of the ToxPanel website is to offer a platform to provide access to our liver- and kidney-injury modules and to calculate gene-set activation scores for gene-set analysis using *log*-transformed FC values. The website also allows users to upload their own gene sets or pathways. Figure 3 shows the job submission page with supported input file formats for gene expression data and customized gene sets. For each gene set, the program calculates the z-scores and *p*-values for both the AFC and AAFC methods. If the user provides gene-level *p*-values in the input file, it also calculates the aggregated *p*-value for a gene set, based on Fisher’s probability test (Fisher, 1932).

**FIGURE 4:**
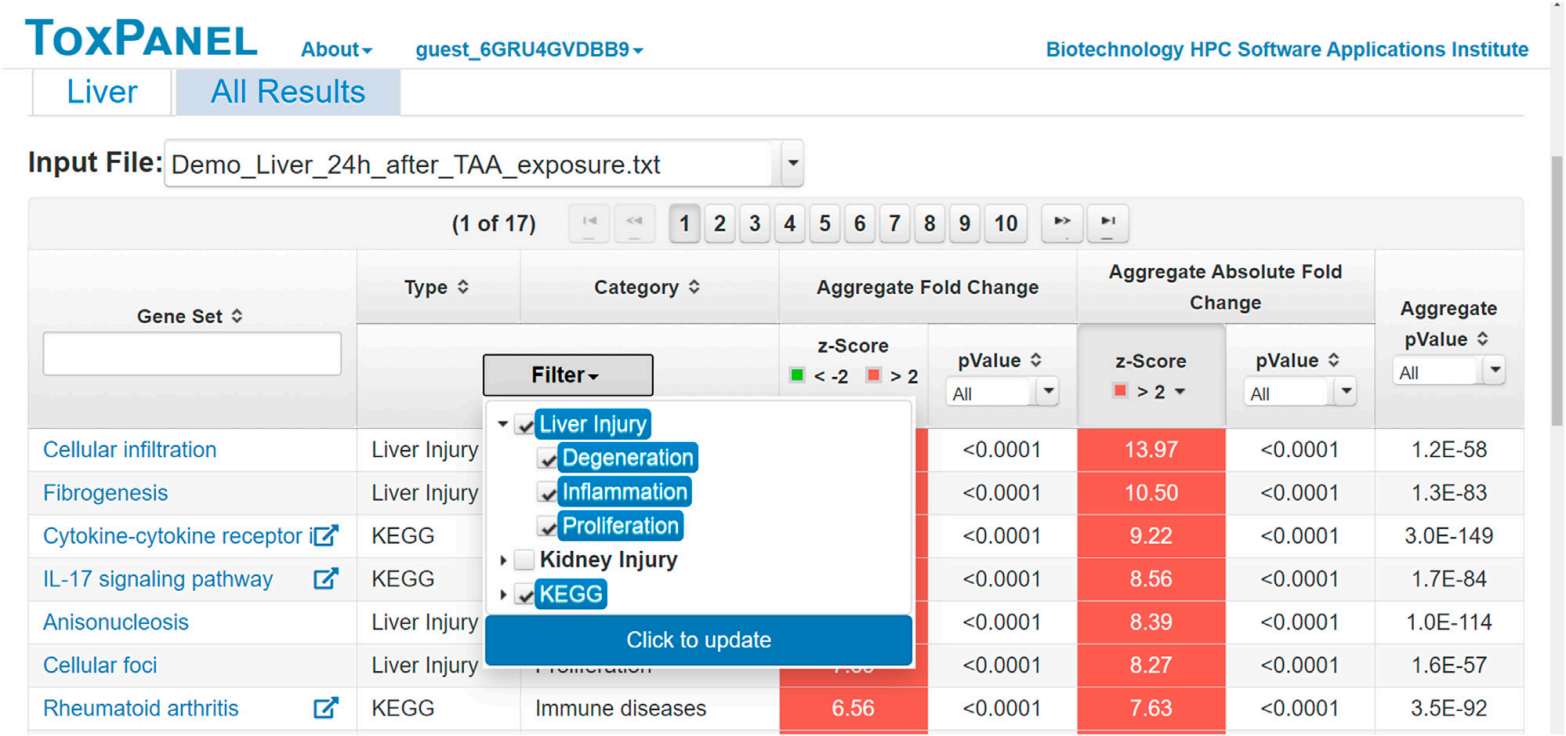
Screenshot of typical results for gene sets activated by the liver toxicant, thioacetamide. Headings: **Gene Set**—name of gene set, injury module, or pathway; **Filter**—type of gene set (e.g., KEGG pathway) displayed when selected; **Aggregate Fold Change z-Score**—positive for an up-regulated gene set and negative for a down-regulated gene set; **Aggregate Absolute Fold Change z-Score**—gene-set activation, as calculated by summing the changes in the expression levels for all genes within the gene set; **Aggregate *p*-Value**—Fisher’s combined *p*-value for all genes in the gene set.

Users can view all of the results on the ToxPanel website or download them for offline analysis. Figure 4 shows a typical output for changes in gene expression following exposure to thioacetamide. By clicking on the name of a gene set, the user can view the genes in that gene set and their corresponding FC values. This is useful for identifying the main genes contributing to a gene set. For each KEGG pathway, we offer a link to its webpage. The main results are shown under the headings of **Aggregate Fold Change** and **Aggregate Absolute Fold Change**. We display both the z-score and *p*-value for each gene set so that users can easily identify significantly activated gene sets. In the example shown in Figure 4, the gene sets are ranked by the z-score of the AAFC method. The top-ranked gene set is *Cellular infiltration* for liver injuries, with an AAFC z-score of 13.97.

In this paper, we introduced ToxPanel as a new tool for assessing liver and kidney injury based on gene expression data. Furthermore, ToxPanel complements existing gene and pathway analysis tools by providing a platform for users to access the AFC and AAFC methods. We have shown that the genes sets provided in ToxPanel can be used for making predictions of liver and kidney injury occurrence in rats before the damage appears (Schyman et al., 2018; Schyman et al., 2020a); and, that rat and human *in vitro* gene expression data correlate with *in vivo* injury observed in rat (Schyman et al., 2019; Schyman et al., 2020b). Thus, ToxPanel can potentially be used in early drug discovery and chemical safety valuations to assess chemical-induced liver and kidney injury from *in vitro* gene expression data.

## Data Availability

The raw data supporting the conclusions of this article will be made available by the authors, without undue reservation.
